# Myocardial arterial spin labeling perfusion imaging with improved sensitivity

**DOI:** 10.1186/1532-429X-16-15

**Published:** 2014-01-27

**Authors:** Hung Phi Do, Terrence R Jao, Krishna S Nayak

**Affiliations:** 1 Department of Physics and Astronomy, University of Southern California, 3740 McClintock Ave, EEB 400, Los Angeles, CA 90089-2564, USA; 2 Department of Biomedical Engineering, University of Southern California, Los Angeles, CA, USA; 3 Ming Hsieh Department of Electrical Engineering, University of Southern California, Los Angeles, CA, USA

**Keywords:** Arterial spin labeling, Myocardial perfusion, Physiological noise, Sensitivity, Parallel imaging

## Abstract

**Background:**

Myocardial arterial spin labeling (ASL) is a noninvasive MRI based technique that is capable of measuring myocardial blood flow (MBF) in humans. It suffers from poor sensitivity to MBF due to high physiological noise (PN). This study aims to determine if the sensitivity of myocardial ASL to MBF can be improved by reducing image acquisition time, via parallel imaging.

**Methods:**

Myocardial ASL scans were performed in 7 healthy subjects at rest using flow-sensitive alternating inversion recovery (FAIR) tagging and balanced steady state free precession (SSFP) imaging. Sensitivity encoding (SENSE) with a reduction factor of 2 was used to shorten each image acquisition from roughly 300 ms per heartbeat to roughly 150 ms per heartbeat. A paired Student’s *t*-test was performed to compare measurements of myocardial blood flow (MBF) and physiological noise (PN) from the reference and accelerated methods.

**Results:**

The measured PN (mean ± standard deviation) was 0.20 ± 0.08 ml/g/min for the reference method and 0.08 ± 0.05 ml/g/min for the accelerated method, corresponding to a 60% reduction. PN measured from the accelerated method was found to be significantly lower than that of the reference method (p = 0.0059). There was no significant difference between MBF measured from the accelerated and reference ASL methods (p = 0.7297).

**Conclusions:**

In this study, significant PN reduction was achieved by shortening the acquisition window using parallel imaging with no significant impact on the measured MBF. This indicates an improvement in sensitivity to MBF and may also enable the imaging of subjects with higher heart rates and imaging during systole.

## Background

Arterial spin labeling (ASL) is a quantitative, contrast-free MRI technique for measuring tissue perfusion. It is most commonly used in the brain for the clinical quantification of cerebral blood flow in cerebrovascular disease and neuro-oncology [1-3]. ASL outside the brain is an immature technology but shows great promise in the heart. Several groups have been able to use ASL to measure myocardial blood flow (MBF) in both animal models [4-9] and humans [10-13]. Myocardial ASL has even been demonstrated to be compatible with pharmacological stress testing and is able to detect clinically relevant increases in MBF with vasodilation [14,15], making it a potential diagnostic tool for detecting ischemic heart disease.

However, myocardial ASL perfusion imaging faces several challenges. The ASL signal has low sensitivity to blood flow and only produces a 1-8% signal change under normal physiological myocardial blood flows of 0.5-4 ml/g/min [16]. ASL also requires the subtraction of multiple image pairs and is particularly sensitive to imperfect subtraction caused by either respiratory or cardiac motion. In this study, we aim to reduce physiological noise (PN), which refers to temporal fluctuations in the ASL signal. We hypothesize that motion within the acquisition window is a major contributor to PN, which can be reduced by accelerating the image acquisition using parallel imaging (PI). We chose parallel imaging for acceleration because of its widespread clinical adoption over the past decade and its well-understood noise and artifact behavior.

In this study, we compare the performance of an accelerated myocardial ASL method using rate-2 sensitivity encoding (SENSE) [17] with a reference, unaccelerated myocardial ASL method [12] in healthy volunteers at rest.

## Methods

### Imaging methods

**Figure 1:**
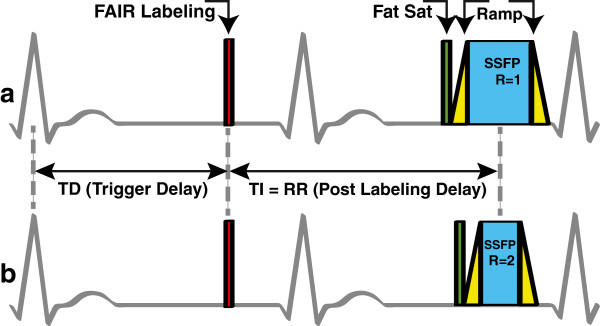
**Schematic of the myocardial ASL pulse sequence. (a)** Reference method [12] and **(b)** accelerated method, using rate-2 SENSE acceleration. The pulse sequence consists of a FAIR labeling pulse (red), a fat saturation pulse (green), a Kaiser-Bessel ramp up (yellow), balanced SSFP image acquisition (blue), and a Kaiser-Bessel ramp down (yellow). The FAIR labeling inversion pulse is triggered to occur at mid-diastole. The center of the acquisition window is set to occur precisely one heartbeat after the tagging pulse. These diagrams are to scale for a heart rate of 60 beats per minute.

All experiments were performed on a Signa Excite HDxt 3 Tesla scanner (GE Healthcare, Waukesha, WI, USA) with an eight-channel cardiac receiver coil. Myocardial ASL was performed on a single short axis middle ventricular slice using flow-sensitive alternating inversion recovery (FAIR) [18,19]. The pulse sequence shown in Figure 1 is composed of either a slab selective inversion pulse for control images or a global inversion pulse for tagged images, a fat saturation module, a 19-TR Kaiser-Bessel ramp up of flip angles to minimize transients [20], a snapshot balanced steady state free precession (b-SSFP) image acquisition, and a 19-TR Kaiser-Bessel ramp down of flip angles.

The reference ASL scan was implemented as described previously by Zun *et al.*[12,15]. All image acquisitions had 3.2 ms TR, 1.5 ms TE, 50˚ prescribed flip angle, 62.5 kHz receiver bandwidth, and 24–26 cm isotropic field-of-view (FOV). Slice thickness was 10 mm, and the selective inversion slab for control images was 30 mm thick to ensure that the imaging slice was within the inversion slab. The reference ASL scans had a 96 × 96 matrix size resulting in an acquisition window of 307 ms. This was preceded by a 10 ms fat saturation module and a 60 ms Kaiser-Bessel ramp up of flip angles and followed by a 60 ms Kaiser-Bessel ramp down of flip angles. The total imaging window of the reference method was 440 ms.

The accelerated ASL scan used sensitivity encoding (SENSE) [17] with a reduction factor of 2 to shorten the acquisition window to 153 ms, as shown in Figure 1. This was also preceded by a 10 ms fat saturation module and a 60 ms Kaiser-Bessel ramp up, and followed by a 60 ms Kaiser-Bessel ramp down. The total imaging window of the accelerated method was 286 ms. We hypothesized that motion during the long acquisition window was a major source of physiological noise and could be reduced by shortening the acquisition window from 307 ms to 153 ms.

Breath-holds and cardiac triggering were used to minimize respiratory and cardiac motion, respectively. The FAIR labeling inversion pulse was timed to occur at mid-diastole through plethysmograph gating (PG). Mid-diastole was estimated to be at 77% of the RR interval duration [21] and the PG trigger delay was set to this value minus 200 ms to account for circulation time from the R-wave to the fingertip [22]. The center of the acquisition window was set to occur one heartbeat (TI = 1RR) after the FAIR labeling pulse. Control and tagged images were acquired in the same breath-hold.

The ASL protocol was comprised of 7 breath-holds and took roughly between 2–3 minutes. In the first breath-hold, baseline and noise images were acquired in 2 seconds. The baseline and noise images were later used for estimating the blood equilibrium magnetization (M_0_) and thermal noise (TN), respectively. Six pairs of control and tagged images were acquired for spatial temporal averaging in the following six 12-second breath-holds. There was a 6-second waiting period between control and tagged images within each breath-hold. The protocol was the same for the reference and accelerated scans except the acquisition window was shortening by using rate-2 SENSE in the accelerated scan.

### Experimental methods

This study included 7 healthy subjects (6 males, 1 female, age 22 – 29 years, mean age = 25 years). The University of Southern California Institutional Review Board approved the study protocol and informed consent was obtained from all subjects. The scan protocol started with a localization scan and identification of a middle short axis slice [23]. A baseline image, an image without the FAIR labeling pulse, was acquired to ensure the slice was prescribed properly without banding artifacts over the myocardium. If banding artifacts were present on the myocardium, a frequency scout scan was performed. The offset frequency that resulted in no banding artifact in the region of interest (left ventricular myocardium) was recorded and used for subsequent scans. A fully sampled baseline image with no banding artifact was then acquired in a 1-second breath-hold to estimate the coil sensitivity map. The reference ASL scan was performed before the accelerated ASL scan in all subjects.

### Data analysis

MBF, PN, and TN were calculated in the same way as in prior work by Zun *et al.*[12,15]. All data processing was performed in MATLAB (Mathworks, Natick, MA). After image reconstruction, the myocardium was manually segmented and resampled into polar coordinates using a spatio-temporal averaging filter [24]. MBF quantification was derived from Buxton’s general kinematic model [25].

$$\mathrm{MBF}=\frac{C-T}{2M_{0}\;\cdot \;\mathrm{TI}\;\cdot \;\exp\;\left(-\mathrm{TI}/T_{1}\right)},$$

where C, T, and M_0_ refer to the mean myocardial signal in the control, tagged, and baseline images, TI represents the post labeling delay time and was equal to the R-R interval, and T_1_ is the longitudinal relaxation time of blood, which was assumed to be 1650 ms [26].

The size of the spatial filter and the number of resampled segments could be freely chosen. Global MBF quantification was performed with a filter size of 2π and a single segment. Regional MBF quantification was performed with a filter size of π/3 and 6 segments [23]. Septal MBF quantification was performed using a filter size of 2π/3 with 3 segments. This measurement was performed in order to compare these data with a prior analysis of physiological noise [12]. A paired Student’s *t*-test was used to compare MBF and PN between the reference and accelerated ASL methods. Agreement between regional MBF measured from the two methods was also assessed by Bland-Altman plot.

## Results

**Figure 2:**
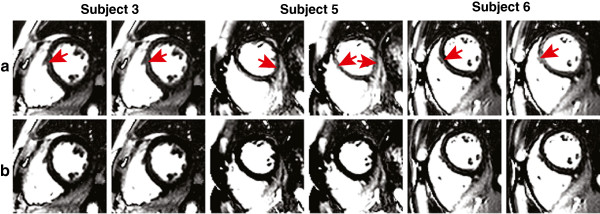
**Control images from three representative subjects. (a)** Reference method and **(b)** accelerated method, using rate-2 SENSE acceleration. All images are windowed identically. Motion artifacts, identified by red arrows, are visible in the images acquired using the reference method whereas SENSE accelerated images showed significantly less motion artifacts.

Representative control images acquired from the reference method are shown in Figure 2a; those acquired from the accelerated method are shown in Figure 2b. There was no visible residual aliasing in the SENSE accelerated images. Motion artifacts were seen in several images acquired from the reference method, as indicated by red arrows whereas SENSE accelerated images showed significantly less motion artifacts. It is worth noting that the per-pixel SNR of baseline images was 126 and 90 for the reference and accelerated acquisitions, respectively. The SNR of baseline images was high in both cases, and this is not expected to be a limitation of the approach.

**Figure 3:**
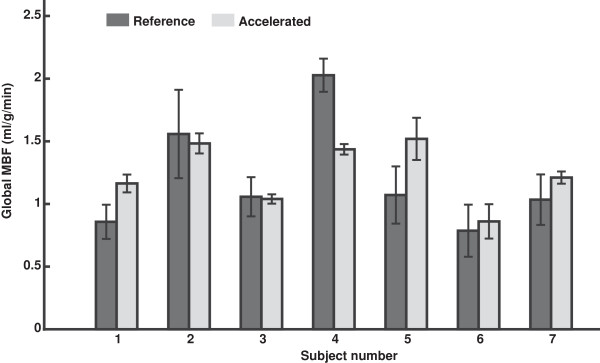
**Measurements of global MBF from all subjects.** (dark gray) Reference method and (light gray) accelerated method, using rate-2 SENSE. Error bars indicate physiological noise standard deviation. No significant difference was found between global MBF measured from the two techniques (p = 0.7297). The mean and standard deviation of PN across all subjects was 0.20 ± 0.08 ml/g/min from the reference method and 0.08 ± 0.05 ml/g/min for the accelerated method, corresponding to a 60% reduction. Physiological noise from the accelerated method was significantly lower than that from the reference method (p = 0.0059).

Figure 3 shows global MBF measured from the reference (dark gray) and accelerated (light gray) methods. Error bars reflect one standard deviation of measured physiological noise. The mean and standard deviation of PN across all subjects was 0.20 ± 0.08 ml/g/min from the reference method and 0.08 ± 0.05 ml/g/min for the accelerated method, corresponding to a 60% reduction. PN measured from the accelerated method was found to be significantly lower than that from the reference method (p = 0.0059). The mean and standard deviation of thermal noise (TN) across all subjects was 0.0175 ± 0.0040 ml/g/min for the reference method and 0.0241 ± 0.0045 ml/g/min for the accelerated method, and the average standard deviation increase was 39% (expected based on the shortened readout time and g-factor losses). The mean and standard deviation of global MBF measured with the reference method were 1.20 ± 0.44 ml/g/min, and measured with the accelerated ASL method were 1.24 ± 0.25 ml/g/min. There was no significant difference between global MBF measured from the two techniques (p = 0.7297). The measured global MBF range of 0.79-2.03 ml/g/min for the reference method and 0.86-1.52 ml/g/min for the accelerated method are consistent with PET literature values 0.73-2.43 ml/g/min [27].

Table 1 compares the accelerated and reference ASL methods. A significant difference was found between global, septal, and regional PN between the two methods with p-values of 0.0059, 0.0002, and 0.0001, respectively. There was no significant difference between global, septal, and regional MBF with p-values of 0.7297, 0.6806, and 0.5353, respectively. Septal MBF and septal PN values for the reference method were comparable to results reported in Ref. [12], which were septal MBF = 1.36 ± 0.38 ml/g/min and septal PN = 0.23 ± 0.12 ml/g/min

**Table 1:** Comparison between the reference and accelerated ASL methods

	**Global**		**Septal**		**Regional**	
	**MBF**	**PN**	**MBF**	**PN**	**MBF**	**PN**
**Reference method**	1.20 ± 0.44	0.20 ± 0.08	1.23 ± 0.56*	0.28 ± 0.11*	1.22 ± 0.68	0.34 ± 0.21
**Accelerated method**	1.24 ± 0.25	0.08 ± 0.05	1.28 ± 0.44	0.17 ± 0.06	1.28 ± 0.46	0.21 ± 0.11
**p-value**	**0.7297**	**0.0059**	**0.6806**	**0.0002**	**0.5353**	**0.0001**

Values are reported as mean ± standard deviation across all subjects in ml/g/min. Values indicated by (*) are comparable to the results reported in Ref. [12].

**Figure 4:**
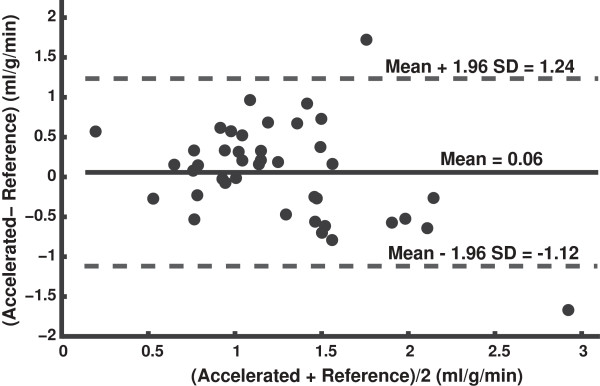
**Bland-Altman plot comparing regional MBF (6 segments per subject) measured from the reference and accelerated ASL methods.** No significant bias was found between regional MBF measurements by the two methods.

Figure 4 shows a Bland-Altman plot comparing regional MBF (6 segments per subject) measured from the reference and accelerated method. There was no significant bias in regional MBF measurements using the accelerated method.

## Discussion

This study demonstrates that PN in myocardial ASL can be significantly reduced by shortening the acquisition window using SENSE, which supports our hypothesis that motion during image acquisition was a dominant source of PN. Figure 2 shows that motion artifacts from control images in the reference method are significantly smaller after shortening the image acquisition window to roughly 150 ms per cardiac cycle. We suspect that motion during the acquisition window causes inconsistencies amongst reconstructed pairs of control and tagged images, which leads to temporal fluctuations in measured MBF for the reference method. Shortening the acquisition window using parallel imaging effectively decreased these likelihood of such inconsistencies, which in turn reduced PN by roughly 60%. The PN reduction corresponds to a 158% increase in temporal SNR (tSNR). An increased tSNR directly translates to improved sensitivity to MBF. Furthermore, MBF measured using the accelerated method was comparable to those from the reference method. There was no significant difference found between global MBF (p = 0.7297) and regional MBF (p = 0.5353) measurements from the two methods.

It may seem counterintuitive that the use of parallel imaging could increase tSNR, when it is known that SNR decreases. Noise analysis from this study revealed that measured TN increased from 0.0175 ± 0.0040 ml/g/min to 0.0241 ± 0.0045 ml/g/min after accelerating by rate 2. While PN decreased from 0.20 ± 0.08 ml/g/min to 0.08 ± 0.05 ml/g/min after acceleration. There was an overall reduction in total noise because the increase in TN from the shortened acquisition window and g-factor losses was much smaller than the decrease in PN from more consistent reconstructions of control and tagged image pairs. This suggests that it may be possible to reduce noise further by pushing the acceleration until the incremental decreases in PN are matched by equal increases in TN.

Although this study only included healthy subjects at rest, one would expect the accelerated ASL method to also improve sensitivity of myocardial ASL in patient cohorts at both at rest and stress. Zun *et al.*[15] reported that global PN from 16 “normal” patients was 0.64 and 1.36 at rest and stress, respectively, which is roughly 3 times higher than that measured from healthy subjects, suggesting an even greater need for PN reduction.

Shortening the acquisition window using SENSE may have the additional benefits of allowing imaging of subjects with high heart rates (>90 beats per minute) and image during systole (<100 ms acquisition window). In subjects with high heart rates, diastole is shorter and faster image acquisition may help reduce cardiac motion artifacts. Likewise, systole is shorter than diastole and may also benefit from the same type of accelerated image acquisition. In addition, the use of a shorter acquisition window per slice may allow the spatial coverage to be extended to 2 or more slices per heartbeat, following a single FAIR label.

One important drawback of SENSE is that when the FOV is smaller than the signal-producing region, SENSE is unable to completely unwrap all aliasing and can lead to residual artifacts at the center of the reconstructed images. Generalized autocalibrating partially parallel acquisition (GRAPPA) [28] is a natural alternative and has been shown to be more robust when the FOV is smaller than the object [29]. Rapid imaging methods based on partial Fourier and/or constrained reconstruction may also be appropriate and remain to be explored.

Parallel imaging has been extensively used in first pass CMR perfusion imaging to improve spatio-temporal resolution and spatial coverage [30]. The primary side effect associated with its use is reduced SNR based on the shortened readout time and g-factor losses. Compared to first-pass methods, cardiac ASL methods (based on apparent-T1 or signal subtraction) are limited by temporal SNR. This is a fundamental difference. Many T1-based methods and signal subtraction methods rely on long image acquisition windows, on the order of 30% of the RR interval, and it is conceivable that these methods will experience an increase in temporal SNR by using parallel imaging, as was demonstrated in this study.

There are several inherent assumptions associated with this particular ASL method. First, we assume a stable heart rate within each breath-hold. Heart rate variability within a breath hold may result in control and tagged images being acquired at slightly different cardiac phase. Second, we use baseline image intensity as a surrogate for M_0_ and assume the blood tissue partition coefficient to be one. In brain ASL, M_0_ is commonly estimated by scaling local tissue with the blood tissue partition coefficient, where the local tissue signal is acquired from a proton density weighted scan. Third, we assume perfect inversion efficiency for all inversion pulses. Based on our own *in-vivo* measurements the efficiency is consistently above 94%. Note that the inversion slab thickness of 3 cm does not introduce quantification errors when using a 1 R-R labeling delay, because this does not leave enough time for unlabeled blood within the LV blood pool to perfuse the myocardium. The inversion thickness would, however, require compensation when using longer labeling delays [8].

## Conclusions

By using parallel imaging to shorten the acquisition window of human myocardial ASL scans, we have demonstrated a significant reduction in physiological noise, with no significant change in measured MBF. The reduction in PN provides more temporally consistent measurements of MBF and improves the sensitivity of myocardial ASL to MBF.

## Consent

Written informed consent was obtained from all seven subjects for the publication of this report and any accompanying images.

## Abbreviations

ASL: Arterial spin labeling; FAIR: Flow-sensitive alternating inversion recovery; MBF: Myocardial blood flow; PN: Physiological noise; TN: Thermal noise; tSNR: Temporal signal to noise ratio; PI: Parallel imaging; SENSE: Sensitivity encoding; GRAPPA: Generalized autocalibrating partially parallel acquisition; TD: Trigger delay time; TI: Inversion time or post labeling delay time; RR: R-wave to R-wave interval; FOV: Field of view; ROI: Region of interest.

## Competing interests

The authors declare that they have no competing of interests.

## Authors’ contributions

HPD designed and implemented the pulse sequences, performed the experiments, performed all data analysis, and drafted the manuscript. TRJ contributed to data analysis methods and revised the manuscript. KSN participated in experiment design, supervised the performance and revised the manuscript. All authors participated in discussion, read and approved the final manuscript.
